# The Impact of Disease Duration on Microcirculatory Dysfunction in Young Patients with Uncomplicated Type 1 Diabetes

**DOI:** 10.3390/biomedicines12051020

**Published:** 2024-05-06

**Authors:** Jolanta Neubauer-Geryk, Melanie Wielicka, Magdalena Hoffmann, Małgorzata Myśliwiec, Leszek Bieniaszewski

**Affiliations:** 1 Clinical Physiology Unit, Medical Simulation Centre, Medical University of Gdańsk, 80-210 Gdańsk, Poland; melanie.wielicka@gmail.com (M.W.); lbien@gumed.edu.pl (L.B.); 2 Diabetology Outpatient Clinic, Non-Public Health Care Institution SAFMED, 83-000 Pruszcz Gdański, Poland; magda2601@poczta.onet.pl; 3 Department of Pediatrics, Diabetology and Endocrinology, Medical University of Gdańsk, 80-211 Gdańsk, Poland; malgorzata.mysliwiec@gumed.edu.pl

**Keywords:** skin microcirculation, capillaroscopy, transcutaneous oxygen pressure, endothelium dysfunction, type 1 diabetes mellitus, children and adolescents

## Abstract

This study aimed to evaluate the earliest changes in the structure and function of the peripheral microcirculation using capillaroscopy and transcutaneous oxygen pressure measurement in children and adolescents with type 1 diabetes mellitus at baseline and during post-occlusive reactive hyperemia (PORH) in the function of diabetes duration. Sixty-seven patients with type 1 diabetes mellitus (T1D), aged 8 to 18 years, and twenty-eight age- and sex-matched healthy subjects were included in the analysis. Diabetic patients were divided into subgroups based on median disease duration. The subgroups differed in chronological age, lipid levels, and thyroid hormones. Capillaroscopy was performed twice: at baseline and then again after the PORH test. Transcutaneous oxygen pressure also was recorded under baseline conditions during and after the PORH test. Comparison of capillaroscopy and transcutaneous oxygen pressure parameters at rest and after the PORH showed no statistically significant difference between the subgroups. This remained true after adjusting for variables that differentiated the two subgroups. However, in the group of patients with long-standing diabetes, significant negative correlations were observed between the Coverage value after the PORH test and capillary reactivity with TcPO_2__zero (biological zero). Significant positive correlations were also found between distance after the PORH test and TcPO_2__zero. The results of our study indicate that in patients with a shorter duration of diabetes, the use of multiple tests provides a better characterization of the structure and function of microcirculation because the onset of dysfunction does not occur at the same time in all the tests.

## 1. Introduction

The clinical course of type 1 diabetes (T1D) has significantly changed in recent years. Advances in research and medical therapies have made that the 10-year duration of diabetes is not the time for diabetic complications to become evident.

The characteristic microvascular complications of type 1 diabetes, such as retinopathy, neuropathy, or nephropathy, are the result of impaired endothelial function. All these complications represent structural capillary changes and progressive deterioration of microvascular function. Therefore, contemporary diabetology seeks to evaluate microcirculation prior to the appearance of clinical complications.

Clinicians continue to express interest in non-invasive studies of microvasculature as a means to detect early signs of microvascular dysfunction. However, only limited tools are currently available. There are a number of non-invasive techniques available to assess the microcirculation [1]. Capillaroscopy [2,3,4,5,6,7,8] and videocapillaroscopy [9,10,11], laser Doppler flowmetry [12,13,14], thermography [15,16], and transcutaneous oxygen measurements [17,18,19,20,21,22,23] are of recognized clinical value. Therefore, any emerging methods for detecting early signs of microcirculatory changes should be evaluated, and if plausible, the standard for use should be established.

The physiologic relationship between blood and capillaries is described by the Fahraeus–Lindqvist effect [24]. Namely, under certain conditions, a reduction in the apparent viscosity of blood flowing in vessels with a diameter below 0.3 mm in diameter leads to the formation of an erythrocyte-free layer. These effects are related to the presence of an endothelial layer. This layer is approximately 0.5–1.0 μm wide and consists of a glycocalyx. It has been shown to have a significant effect on microvascular hemodynamics, primarily on hematocrit and flow resistance. This layer is also involved in the regulation of vascular tone, endothelial sensitivity to mechanical factors, interaction of endothelial cells with cells of the immune system, modulation of diffusion processes, and the processes of coagulation and angiogenesis [25].

The density of the capillary network varies between different tissues, depending on the functional state of the organ. The capillary wall consists of an endothelial layer surrounded by a basal membrane with some smooth muscle fibers. There is some variability in the structure of the wall: in a large capillary, several endothelial cells may line the lumen, bordering each other; in a small capillary, there may be only a single layer of cells surrounding the lumen. The vascular endothelium plays a major role in autoregulation through the production of numerous mediators. Among the most important vasodilators produced by endothelial cells are nitric oxide and prostacyclin. Vasoconstrictors include endothelin and vascular endothelial growth factor. Endothelial injury, including hyperglycemia, hypertension, and inflammation, results in organ failure.

The proper microcirculatory function is necessary for effective hemostasis [26]. According to the results of Ijzerman’s study [27], skin microvasculature may be suitable to study the relationship between cardiovascular risk factors and microvascular function. In fact, the earliest symptoms of cardiovascular diseases appear in microcirculation, especially those related to inflammation [9,28]. Microvascular abnormalities are found in conditions like diabetes [3,29], renal disorders [30], cardiovascular disorders [31,32,33] like hypertension [34,35,36] or systemic connective tissue disorders [2], septic shock [37], and hypoxia [38]. They primarily lead to flow abnormalities, which are associated with impaired tissue oxygenation and organ damage and hurt prognosis [33,36].

At rest, only about 25% of the blood vessels in microcirculation are active [36,39]. One of the most commonly used tests to evaluate peripheral microvascular function is post-occlusive reactive hyperemia (PORH). It allows the observation of a transient increase in cutaneous perfusion immediately after the release of an arterial occlusion. This is referred to as reactive post-occlusion congestion, which involves shear stress and its effects [40]. The test demonstrates capillary recruitment during occlusive reactive congestion, which involves endothelium-dependent precapillary vasodilation [41,42] mediated by axonal reflex, an endothelium-derived hyperpolarizing factor. PORH involves both myogenic and metabolic factors [43]. The role of nitric oxide (NO) in the test has been controversial and is now thought to have no role in the vasoconstriction response [43,44,45,46]. The changes in microcirculatory blood flow during the active congestion phase of the test can be demonstrated with a curve. This test primarily evaluates peak perfusion, which is represented by the area under the curve. During the PORH test, capillary recruitment takes place [47].

Cutaneous microcirculation is responsible for maintaining normal body temperature and is very sensitive to local changes in temperature. Transcutaneous oxygen pressure (TcPO_2_) applies this phenomenon to test microcirculatory reactivity in response to temperature increases. In the thermal congestion test, local blood flow is increased by heating the skin. An increase in localized skin perfusion is the result of vasodilation and is directly proportional to skin temperature. The degree of vasodilation peaks when the local skin temperature reaches approximately 44 °C and is maintained for at least 20 min. The vascular response during this test is biphasic. The first phase, a rapid rise in flow, occurs within the first 10 min and is associated with sensory nerve involvement. The second phase, a plateau lasting approximately 20–30 min, is mediated primarily by nitric oxide [13,39]. In a meta-analysis, Fuchs et al. [13] observed a lower microvascular response to heat stress in diabetic patients than in healthy subjects. They also noted that diabetic patients with microvascular complications had an impaired vascular response compared to patients without microangiopathy.

Despite significant advances in the diagnosis, prevention, and treatment of type 1 diabetes in recent decades, chronic diabetes complications in the form of microvascular and macrovascular complications remain a major cause of disability, reduced quality of life, and inability to work.

Non-invasive techniques, especially those implementing provocative stimuli, are the preferred method for evaluating microcirculation. We designed a study to evaluate the structure and function of peripheral microcirculation in young patients with type 1 diabetes with the use of capillaroscopy and transcutaneous measurement of partial pressure of oxygen at rest and following post-occlusion active congestion. Additionally, we analyzed how disease duration, the degree of metabolic compensation of diabetes, and selected biochemical markers were determined.

## 2. Materials and Methods

### 2.1. Study Group

The study group included 67 patients with type 1 diabetes (T1D), 29 boys and 38 girls, with a mean age of 14.7 (10.9–18) years treated at the Department of Pediatrics, Diabetology, and Endocrinology at the University Clinical Center in Gdańsk who met the type 1 diabetes diagnostic criteria according to the International Society of Child and Adolescent Diabetes [48]. The median age at onset of diabetes was 10.4 years, and the median duration of diabetes was 4.65 years. The control group included 28 subjectively healthy volunteers (15 boys and 14 girls) aged 14.5 (±2) years (Table 1).

Subjects for the control group were recruited from the families of the patients included in the study.

Exclusion criteria for the study were micro- and macroangiopathic complications, acute complications of diabetes, abnormal levels of TSH and free thyroxine, autoimmune thyroiditis, systemic diseases such as rheumatoid arthritis, psoriasis, and statin use. Exclusion of diabetic retinopathy was based on an ophthalmoscopic evaluation of the fundus after pupil dilation by an ophthalmologist according to the American Diabetes Association criteria [49]. Diabetic neuropathy was diagnosed based on subjective and objective neuropathy symptoms [50]. Diabetic nephropathy was diagnosed based on the current determination of albumin levels in a collected urine sample and albuminuria tests during the last 6 months before the study.

Severe hypoglycemia was defined as an episode of blood glucose < 54 mg/dL requiring intervention from another person that occurred within a year prior to conducting the survey, but no more than one month before the survey. Mild hypoglycemia was defined as an episode of blood glucose < 54 in the month before the survey that did not require intervention [51].

The subjects of the control group did not have any disorders of carbohydrate metabolism.

A detailed medical history was obtained from the subjects at baseline. An experienced pediatrician performed the examination. Informed consent was obtained from the subjects after a detailed explanation of the purpose and conduct of the study.

The research methodology used was approved by the Independent Bioethics Committee for Scientific Research at GUMed (decisions NKBBN/277/2014 of 8 July 2014 and NKBBN/277-512/2016 of 5 December 2016).

In the period before the capillaroscopic examination, the resting systolic and diastolic blood pressure values were measured five times with an OMRON HEM-907 automatic sphygmomanometer in a sitting position after at least 10 min of rest.

The study group of 67 patients with diabetes mellitus t.1 (T1D) was not statistically significantly different from 28 control subjects (C) in gender distribution, body mass index, and age. In addition, the study group and the control group did not differ in the distribution of the stage of stage of puberty according to the Tanner scale (*p* = 0.96). Mean systolic and diastolic blood pressure and heart rate at rest showed no statistically significant differences between the study groups.

### 2.2. Evaluation of Microcirculation

Prior to the examination, the patients were asked to avoid cosmetic procedures on the fingers for at least 2 weeks. The examination was performed in a quiet room with a constant temperature of approximately 20 °C, which allowed for 15 min of thermal adaptation. Body temperature was controlled with a contactless thermometer (Novama model NT19) and was within normal range in all patients and controls.

#### 2.2.1. Nailfold Capillaroscopy

**Figure 1:**
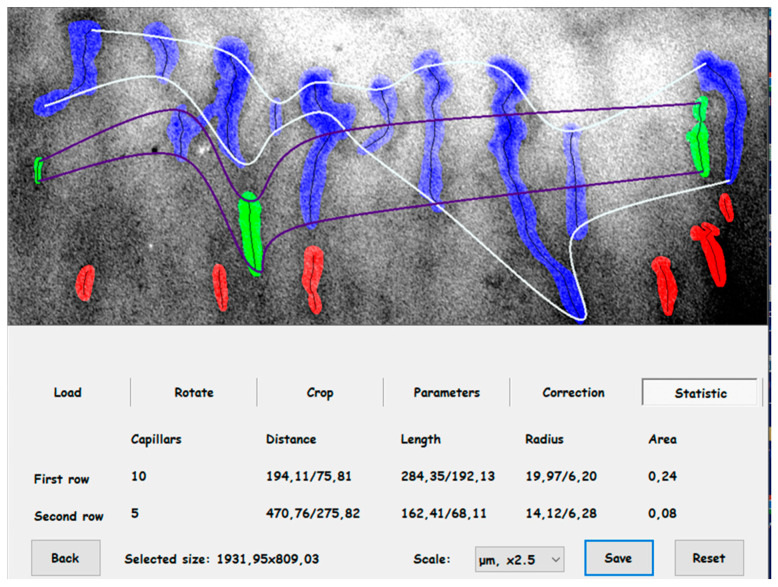
Summary of capillary analysis of one of the test areas of nailfold coverage area and distance analysis.

The capillaroscopic examination was used to evaluate the capillaries of the nail beds of fingers II-V of both upper extremities. Prior to capillaroscopy, the selected fingernail was cleaned and covered with immersion oil to optimize image quality. The capillaroscopic examination was performed in a sitting position with the hands supported so that the palms were free to rest under the capillaroscope. The examination was performed with an OPTA-TECH capillaroscope with two-point illumination, equipped with a 5-megapixel digital camera with 200× magnification. Images were archived on disk using the manufacturer’s standard software (Figure 1).

The capillaroscopic images were analyzed according to a method developed in the Clinical Physiology Laboratory. This method was published in Diabetes Care in 2013 [52].

The mean distance between consecutive capillaries (Distance), the ratio between the area occupied by capillaries, and the total area of the analyzed capillary rows (Coverage) were determined as parameters characterizing the skin microcirculation. These indices were obtained under baseline conditions and after the PORH test. The same areas of the fingernails were analyzed during each exam.

Capillaroscopy during the test was performed twice: after 20 min of rest in a sitting position and after 4 min of active occlusion induced by an arm compression with a sphygmomanometer cuff at a pressure higher than the patient’s systolic pressure by 50 mmHg. Additionally, we have previously studied the reproducibility of capillaroscopy and have described our approach in detail in a separate report [52,53].

Coverage_BASE_—the ratio of the surface area covered by capillaries to the total examined area at baseline,

Coverage_PORH_—the ratio of the surface area covered by capillaries to the total examined area after post-reactive occlusive hyperemia,

∆Coverage_PB_ = Coverage_PORH_ − Coverage_BASE_ (the difference between the values of coverage obtained after and before post-reactive hyperemia)

Capillary reactivity = ∆Coverage_PB_/Coverage_BASE_

Distance_BASE_—mean distance between successive capillaries at baseline,

Distance_PORH_—average distance between successive capillaries after post-occlusion hyperemia

∆Distance_PB_ = Distance_PORH_ − Distance_BASE_ (the difference between the distance values obtained after and before post-occlusive hyperemia).

#### 2.2.2. The Transcutaneous Oxygen Pressure

The transcutaneous oxygen pressure was measured on the patient’s forearm. The sensor was placed on the medial part of the forearm, which was cleaned and free of hair. Transcutaneous oxygen testing was performed with PeriFlux 5000 (Perimed AB, Järfälla, Sweden). TcPO_2_ provides information about the body’s ability to deliver oxygen to tissues based on the amount of oxygen diffusing from the capillaries through the epidermis to the electrode [17,21]. To measure TcPO_2_, sensors contain a pair of polarizing electrodes to determine the current oxygen content of a given volume. The classic oxygen electrode contains a silver-silver anode and a gold or platinum cathode. They are separated by a liquid electrolyte. The electrodes are separated by a polymeric membrane, which allows oxygen to selectively permeate from the area of skin tested [21]. The output current is proportional to tissue oxygen partial pressure [19] and skin blood flow, oxyhemoglobin dissociation, and tissue metabolic activity. The test uses an electrochemical electrode and local heating. A skin temperature of 43 °C was chosen to achieve local congestion [54].

**Figure 2:**
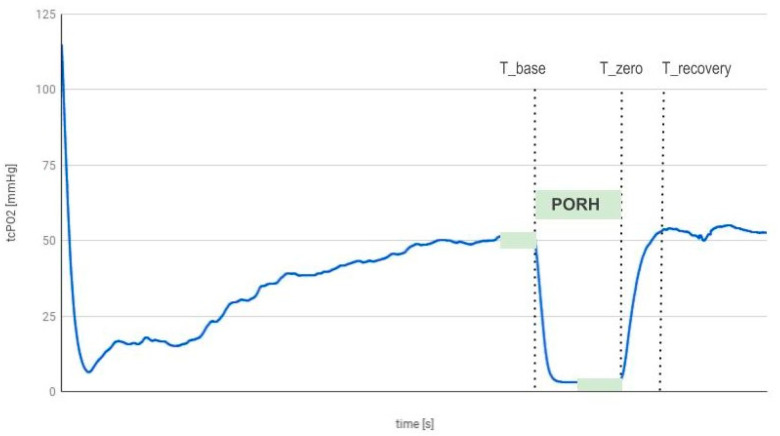
Recording of the TcPO_2_ measurement during the PORH test.

TcPO_2_ was recorded after at least 20 min of rest, during occlusion, and after cuff deflation (Figure 2).

The test data were presented as a graph.

The analysis of the curve included the identification of specific points, i.e., T_base for the moment when occlusion began, T_zero as the moment when occlusion ended, and T_recovery as the moment when recovery was achieved.

The following parameters have been determined:

- TcPO_2_—base–the mean value of TcPO_2_ within 60 s before T_base;
- TcPO_2_—zero–the mean value of TcPO_2_ within 60 s before the T_zero;
- Slope—the ratio of TcPO_2__diff and TTR:

- TcPO_2_—diff–the difference between TcPO_2__base and TcPO_2__zero;
- TTR—time to reach baseline value after occlusion (T_recovery-T_zero).

### 2.3. Laboratory Analysis

After an overnight fast, blood samples were obtained between 7 and 9 a.m. The serum was separated from venous blood within 30 min of collection and stored frozen at −80 °C for up to three months before analysis. The same sample of blood was used for all of the measurements. HbA1c, with a normal range of 3.0 to 6.0%, was measured by an immunoturbidometric method using the Unimate 3 set (Hoffmann-La Roche AG, Basel, Switzerland). Fasting glucose was measured using an enzymatic assay (Roche Diagnostics GmbH, Mannheim, Germany). An immunochemical system (Beckman Instr. Inc., Galway, Ireland) was used to measure C-reactive protein levels. Total, HDL, LDL cholesterol, and triglyceride levels were measured using Cormay enzymatic kits (Cormay, Lublin, Poland). Urinary albumin excretion was measured through an immunoturbidometric assay. Tina-quant was used (Boehringer Mannheim GmbH, Mannheim, Germany). The creatinine level in the serum was measured through the CREA assay system (Boehringer Mannheim GmbH).

### 2.4. Statistical Analysis

Statistical analysis was performed using STATISTICA version 13.1 statistical software from StatSoft Inc., Tulsa, OK, USA; license CSM GUMed JPZP5077539317AR-H.

The Shapiro–Wilk test was used to analyze the distribution of continuous variables. Values were expressed as either median and range or mean and standard deviation. The Wilcoxon test was used to compare two related measurements before and after testing.

Correlations were assessed using Spearman’s rank correlation test for all continuous variables.

Mann–Whitney U test was used to compare two dependent groups, while Kruskal–Wallis ANOVA test, with a selected post hoc test, was used to compare three independent groups. A comparison of skin microcirculation parameters with age, and duration of diabetes was performed using ANOVA test. The χ2 test was used to compare the percentage of gender, the stage of sexual maturity as assessed by the Tanner scale, and the number of mild and severe hypoglycemic episodes.

The ANCOVA test was used to compare the groups with regard to the studied parameters and concomitant variables. Fisher’s test was used for the post hoc analysis. A *p*-level of <0.05 was considered statistically significant.

## 3. Results

The analysis of the correlation between age and parameters that describe the state of peripheral microcirculation assessed by capillaroscopy revealed a significant correlation between age and Distance_BASE_ in T1D (r = 0.28, *p* = 0.02). In turn, there was no significant correlation between age and parameters describing transcutaneous oxygen pressure in T1D and control groups.

The T1D group and the control group did not differ in age. To study the effect of the duration of diabetes on the condition of cutaneous microcirculation, the entire study group was divided into two subgroups based on the median duration of diabetes: subgroup D1 (<4.65 years) and subgroup D2 (≥4.65 years). These subgroups varied in terms of age, age of onset, and frequency of mild hypoglycemia. Patients with longer diabetes duration were younger-group D2 developed diabetes at a younger age and had a higher incidence of mild hypoglycemia in this subgroup. The two subgroups and the control group did not differ significantly in terms of gender distribution and BMI.

There were no statistically significant differences in glycated hemoglobin levels between the subgroups of patients with T1D. The concentration of total cholesterol, LDL fraction cholesterol, and triglycerides was significantly higher in subgroup D2 than in the control group (Table 2). Subgroup D2 also had significantly higher triglyceride and LDL cholesterol concentrations than patients in group D1. When metric age was included in the comparative analyses, the above differences between the subgroups of patients with T1D and controls persisted.

There were no significant differences between the subgroups of patients with T1D with regard to creatinine concentration, CRP levels, and albuminuria. There were also no statistically significant differences in creatinine concentration and CRP levels between the T1D subgroups and the control group. On the other hand, there were significant differences in the levels of the thyroid hormones TSH and fT4. Control subjects had significantly lower TSH and higher fT4 concentrations compared to both subgroups D1 (*p* = 0.04; *p* = 0.03) and D2 (*p* = 0.008; *p* = 0.006). Euthyroid status was present in all study patients and controls. Among the subgroups of patients with T1D, there were no significant differences in TSH and fT4 levels. There were no significant differences in TSH and fT4 levels between the subgroups of T1D when age was taken into account in the analysis.

The diabetes and control subgroups were not statistically different with regard to the levels of systolic and diastolic blood pressure and heart rate, even after adjusting for metric age. The distribution of pubertal stages was not statistically different between the control group and the diabetes group (*p* = 0.99), as well as between the control group and the patient subgroups D1 and D2 (*p* = 0.051).

The subgroups of diabetic patients differed in metric age as well as in lipidogram parameters and thyroid hormone levels. Therefore, these variables were also included in the analyses.

The comparison of the parameters characterizing the capillaroscopic image both at rest and after the PORH test did not show any statistically significant differences between the subgroups of patients with T1D that differed in the duration of the disease. There were also no differences between these subgroups after adjusting for the variables differentiating the subgroups. After adjustment for metric age, lipid profile, and thyroid hormones at baseline, PORH test coverage values were significantly higher in the control group than in patients with T1D. There was a statistically significant difference in Coverage_BASE_ values between the control group and subgroup D2 (*p* = 0.06). When the values of capillaroscopy parameters were analyzed after adjusting for age, there was a statistically significant difference in Coverage_PORH_ values between the control group and T1D subgroups. There was a significant reduction in Coverage values in the PORH test in subgroup D1 (*p* = 0.001).

There was a statistically significant difference in TcPO_2__zero values between the control group and subgroup D2, as well as between subgroups of patients with diabetes, D1 and D2. However, after adjustment for metric age, lipid profile, and thyroid hormone levels, there were no significant differences between the studied subgroups with regard to parameters describing transcutaneous oxygen pressure.

In subgroup D1, there was a statistically significant correlation between Distance_PORH_ and HbA_1c_ levels (r = 0.42; *p* = 0.02). In subgroup D2, consisting of patients with a longer duration of disease, there were no significant correlations between capillaroscopic, and metabolic compensation parameters and analyzed biochemical parameters.

In controls, there was a significant positive correlation between TcPO_2__base and HDL cholesterol (r = 0.45, *p* = 0.02) and a negative correlation between TcPO_2__zero and HbA_1c_ (r = −0.48, *p* = 0.01). Subgroup D1 showed a statistically significant correlation between TTR and CRP (r = 0.39, *p* = 0.03) and creatinine levels (r = 0.41, *p* = 0.03). In subgroup D2, a statistically significant correlation was found between TTR and TSH (r = 0.36, *p* = 0.04).

Both total cholesterol and triglycerides were significantly higher in T1D. However, the study groups did not differ significantly in the concentration of the LDL and HDL cholesterol fractions. Patients with type 1 diabetes and controls did not differ significantly in serum creatinine and CRP levels. Patients with diabetes had statistically higher TSH levels and lower fT4 levels than controls. However, In both the control and type 1 diabetes groups, TSH and fT4 levels were within the normal range.

A comparison of the diabetic and control groups showed that Coverage values were significantly higher in the control group both under baseline conditions (Coverage_BASE_) and after the PORH test (Coverage_PORH_). The within-group analysis showed significant changes in Coverage values only in the diabetes group. As a result of the PORH test, Coverage_PORH_ values were significantly reduced compared to Coverage_BASE_.

In the diabetic group, there was also a significant increase in distance (*p* = 0.04) in the PORH test compared to baseline. The parameters characterizing the cutaneous microcirculation did not show any significant differences between the sexes in either the control group or in the group of patients with T1D.

The study group of T1D patients and the control group did not differ statistically significantly in the values of the parameters TcPO_2__base, TTR, and SLOPE. Only the value of TcPO_2__zero was shown to be statistically significantly higher in T1D than in the control group (*p* = 0.007).

The relationship between capillaroscopy parameters and transcutaneous oxygen pressure showed no significant relationship between parameters describing cutaneous microcirculation and parameters characterizing transcutaneous oxygen pressure in the control group. However, in the type 1 diabetes group, we showed a significant correlation between both ∆Coverage_PB_ and capillary reactivity and TcPO_2__base (r = 0.26, *p* = 0.03 and r = 0.27, *p* = 0.03; respectively). In subgroup D1 of patients with diabetes there were no significant correlations between the capillaroscopy and TcPO_2_ parameters, whereas in subgroup D2 there were significant negative correlations between Coverage_PORH_ and capillary reactivity and TcPO_2__zero (r = −0.43, *p* = 0.01; r = −0.37, *p* = 0.03; respectively) and significant positive correlations between Distance_PORH_ and TcPO_2__zero (r = 0.42; *p* = 0.02). We also observed significant negative correlations between Coverage_PORH_ and TTR (r = −0.42; *p* = 0.01).

## 4. Discussion

Clinical complications of diabetes can be considered as progressive deterioration of capillary function and its structure therefore the challenge is to identify abnormalities in the structure and function of the microvasculature that may precede overt clinical vascular complications.

It was planned to evaluate data from non-invasive studies that evaluate easily accessible areas of the microcirculation. So far, most of the studies that use non-invasive methods of evaluating microcirculation have been conducted in adult patients with diabetes.

### 4.1. Capillaroscopic Changes

It is generally accepted that the number of micro- and microvascular diabetes complications is related to disease duration.

#### 4.1.1. Diabetes Duration

Cisło et al. [4] studied a group of young patients with T1D. Changes in cutaneous microcirculation were observed even in young patients. They were independent of the age at onset and worsened with increasing disease duration. Similar findings were reported by other researchers [5,6,10,55,56,57]. In patients with T1D, they showed a correlation between duration of diabetes and the presence of changes in capillaroscopic images. On the other hand, in adults with uncomplicated type 1 diabetes with a disease duration of 10 years, Tibiriçá et al. [58] did not show any significant correlation between the function of cutaneous microvasculature and the duration of diabetes. Based on their study, the authors concluded that although patients with T1D do not have skin capillary thinning of the skin, they do have changes in the cutaneous microcirculation that are characterized by a lack of capillary reserve.

Tooke et al. [59] showed a duration-related disturbance of microcirculatory vascular autoregulation in patients with T1D. In those with less than 1 year of disease duration, the disturbance was mild, whereas in those with more than 10 years of disease duration, the disturbance was significant. In a group with long-term T1D, Trapp et al. [8] showed no changes in the capillary morphology, number, and density compared to a control group. Neubauer-Geryk et al. [52], who evaluated the cutaneous microcirculation with capillaroscopy and L-arginine infusion in a group of adult patients with T1D, found a clear negative correlation between capillary vascular reactivity and the age at onset of diabetes. However, there was no such correlation with the duration of diabetes. The group of patients with a mean age of onset greater than 19 years, i.e. with shorter disease duration, was characterized by lower cutaneous microvascular reactivity than the group of patients with a longer duration of diabetes. Despite some data being available, it is challenging to effectively compare these studies due to differences in methodology.

To evaluate the effect of diabetes duration on microvascular structure and function, we have divided the study group of young patients with diabetes according to median diabetes duration into subgroup D1 (<4.65 years) and subgroup D2 (≥4.65 years). Analyzing the parameters characterizing cutaneous microcirculation, we showed that the subgroups of patients did not differ in the quantitative parameters, both under resting conditions and in response to the active congestion test (PORH). Compared to diabetic young patients, healthy controls had higher capillary density (coverage) both at rest and after the test. After taking into account the parameters differentiating the subgroups, namely age, lipid, and thyroid hormone levels, the differences were even more pronounced. In our study, there was no association between the duration of diabetes and the severity of skin microcirculation changes. We found significantly denser areas (Coverage_BASE_) in the subgroup of patients with shorter duration of diabetes (D1) compared to the control group. The subgroup of patients with longer disease duration (D2) differed from controls in Coverage_BASE_ at the border of statistical significance (*p* = 0.06). In the diabetic group, this may indicate a phenomenon of capillary thinning.

#### 4.1.2. Subject Age and Gender

Several authors [11,60,61,62] have described the effect of age on skin capillary density. They showed a significant reduction in skin capillary density in older subjects in comparison with young subjects [61,62]. Piotto et al. [11] found that capillary density increased significantly with age in healthy children. In our study, metric age did not differ between the groups studied. However, the subgroups of diabetic patients (D1 and D2) differed in metric age. There were also differences in resting capillaroscopy values between the shorter and healthy subgroups (*p* < 0.001) and between the longer and healthy subgroups (*p* = 0.06) after adjusting for metric age, lipid profile, and thyroid hormone levels. We found no effect of gender on the capillaroscopic parameters we studied.

#### 4.1.3. Metabolic Control

Reports published to date on the relationship between skin microvascular parameters and metabolic control in patients with T1D have not been consistent [7,52,58,60,63,64,65]. Some studies have shown that cutaneous microvascular reactivity is independent of metabolic status reflected by HbA_1c_ values [52,58,60,66]. Meanwhile, in a group of adult patients, Jorneskog et al. [64] showed significant differences in capillary flow between patients with poor and good metabolic control regardless of diabetes duration. Other investigators have shown either an increase in baseline perfusion associated with an HbA_1c_ > 7.5% in adults [65] or no difference in baseline perfusion between participants with T1D and control subjects [67]. Additionally, some studies have shown a relationship between increasing HbA_1c_ levels and the degree of skin microcirculation alterations [5,7,55,57,63]. Our study showed that HbA_1c_ levels did not differ between subgroups of patients with diabetes of different durations. Our analysis of the relationship between the parameters describing the skin microcirculation and the HbA_1c_ level showed that only the Distance_PORH_ value correlated with the HbA_1c_ level in the subgroup of patients with shorter disease duration. None of the capillaroscopy parameters correlated significantly with HbA_1c_ levels in any of the studied groups. Similarly, the study by Gasser et al. [68] showed no correlation between the degree of metabolic control in diabetes and capillaroscopic parameters. Neubauer-Geryk [53], in their study of a group of adult patients with T1D, also found no relationship between cutaneous microvascular reactivity and metabolic control reflected by HbA_1c_ levels.

In our study, glycemic control, presented as HbA_1c_, does not seem to reflect the effect of the current glycemic level on cutaneous capillary structure and function. Obtaining more data on the trends in glycemic control during the disease period would add valuable information that could help explain some differences in microcirculation parameters.

A positive correlation between cholesterol levels and the degree of changes in capillaroscopy exams has been demonstrated in the literature [5]. In the present study, we stated that the diabetic group had higher total cholesterol and triglyceride levels compared to the healthy controls. It should also be noted that compared to the shorter duration group and the control group, the longer duration group was characterized by significantly higher levels of LDL cholesterol and triglycerides.

In our study, adjusting for lipid levels allowed us to demonstrate significant differences in capillaroscopy parameters (Coverage_BASE_ and Coverage_PORH_) between groups D1, D2, and the control group (Table 3).

#### 4.1.4. Cutaneous Microcirculation and Microangiopathies

The methods for assessment of cutaneous circulation we used in this study, namely capillaroscopy and transcutaneous oxygen partial pressure measurement, have been repeatedly validated in type 1 diabetic patients with existing microangiopathy. In fact, several studies have demonstrated abnormalities in capillary imaging being associated with clinical manifestations of microangiopathy [69]. However, the reported conclusions have been inconsistent [6,56,68,70]. Kuryliszyn-Moskal has shown an association between changes in capillaroscopy and the occurrence of diabetic microangiopathy [5,6,71]. Moreover, Kuryliszyn-Moskal et al. [6,56] found that the degree of changes in capillary morphology assessed by capillaroscopy correlated with metabolic control and the presence of chronic complications in the course of type 1 diabetes. In a group of 106 patients with type 1 diabetes, capillaroscopic changes were observed in 81% of patients.

In patients with diabetes without microangiopathy, the capillaroscopic image was either normal or included minor changes, while in patients with diabetes diagnosed with microangiopathy, the capillaroscopy image demonstrated widespread, advanced changes [6]. A study by Hosking et al. [63] found more nonvascular areas on capillaroscopy in patients with microalbuminuria. On the other hand, Pazos-Moura et al. [72] found no differences in capillary density between adult patients with diabetes and healthy subjects. In turn, in our study, we found that patients with type 1 diabetes with no microangiopathic complications have significantly lower capillary density at baseline when compared to healthy controls. The subgroups divided based on diabetes duration, however, did not differ in capillary density. Post hoc analysis did not impact these findings.

### 4.2. Transcutaneous Oxygen Pressure Changes

Most of the published studies using transcutaneous oxygen pressure measurement have been performed in type 2 or mixed type 1 and 2 diabetic patients with micro- and/or macroangiopathic complications [73,74,75,76]. Patients with diabetes lasting less than 1 year and without detectable microangiopathic complications differed significantly from controls in TcPO_2_ [22]. Children with type 1 diabetes have been shown to have less reactive skin congestion expressed as TcPO_2_ compared to the control group [23,77]. We also demonstrated improved microcirculatory function after pancreas transplantation in patients with type 1 diabetes [78]. TcPO_2_ was useful in qualifying diabetic patients for permanent spinal cord stimulation for critical limb ischemia. To date, many studies have demonstrated an association between diabetes and microvascular dysfunction [23]. There is some variation in the literature concerning electrode temperature when using transcutaneous oxygen pressure. Values of 37 °C [79], 43.5 °C [73], 44 °C [19] to 45 °C [54,75] have been reported. In our study, we used the temperature of 43℃ [76]. Different locations of the electrodes have also been used: on the dorsum of the foot [19,73,75,76] or the arm or forearm [54]. In Lagerkvist’s study, the TcPO_2_ value obtained in healthy children and adolescents was 81 (65;97) mmHg, whereas, in our study, the TcPO_2_ value recorded on the arms of control subjects at the beginning of the study was 56.3 (18.6;81.1) mmHg. The different positions of the electrodes and the higher temperature used in Lagerkvist’s study (45 °C) may explain the difference between the results obtained.

De Meijer et al. [76] found that diabetic patients with no evidence of peripheral disease or neuropathy had significantly lower TcPO_2_ values on the lower limb compared to those without diabetes. They did find a significant association between TcPO_2_ and duration of diabetes [76]. Ewald et al. [79] conducted a study on children with diabetes under the age of 15. They showed that after two years of diabetes, vascular reactivity was significantly lower than controls. Ewald’s multiple linear regression analysis showed that urine glucose excretion, plasma glucose concentration, glycated hemoglobin, serum triglyceride and total cholesterol concentrations, diabetes duration, and insulin dose per kg body weight explained 54% of the variation in vascular reactivity. Ewald concluded that the usual parameters of carbohydrate control alone could not explain the reduced vascular reactivity in diabetic children. Breuer et al. [22] showed an association between reduced TcPO_2_ and type 1 diabetes. Significant differences were noted even in patients with a disease duration of less than one year. The observations of Breuer support the possibility that functional abnormalities of the microcirculation in patients with diabetes may precede any structural changes. The only study using PORH to assess TcPO_2_ in adult (insulin-dependent) diabetic patients is the study by Railton et al. [80]. Ischemic response in the cuffed arm, as measured by transcutaneous oxygen pressure at 37 °C, was significantly lower in adult diabetic patients with HbA_1c_ 5.6–8.7%. However, they found no correlation between TcPO_2_ and HbA_1c_. A study by Jorgensen et al. [81] in young adults with T1D without overt microangiopathy showed that TcPO_2_ correlated with a degree of glycemic control, and patients with HbA_1c_ ≤ 9.5% had TcPO_2_ comparable to controls. In contrast, significant abnormalities in transcutaneous oxygen pressure measurements were found in patients with HbA_1c_ ≥ 12.5%. In our present study, we did not find differences in TcPO_2_ under basal conditions. However, during PORH we found that TcPO_2__zero was significantly higher in the diabetic group, while during intragroup comparisons it was higher in children with a longer duration of diabetes. However, when differences in age, lipid, and thyroid hormone levels were taken into account, these differences were no longer present.

Analysis of the effect of sex on skin microcirculation, in the study by Orenstein et al. [82] showed that healthy women had a significantly higher TcPO_2_ than men. We found no correlation between gender and TcPO_2_ parameters in the entire study cohort, similar to the study by Rodrigues et al. [83].

Simultaneous assessment of two different vascular territories showed no correlation between parameters describing both vascular beds in either the control or the shorter diabetic subgroup. However, there was a statistically negative correlation between Coverage_PORH_ and capillary reactivity and TcPO_2__zero in the group of patients with a longer duration of diabetes.

### 4.3. Post Occlusive Reactive Hyperemia

For our study, we chose to use capillaroscopy with the use of a non-invasive provocative test in the form of PORH. This is a widely used method for the assessment of microcirculation [58,60,64,84]. The main mechanism involved in the PORH test is shear stress and its consequences [40]. The capillary recruitment observed in the skin during post-occlusive reactive congestion is related to endothelium-dependent vasodilation at the precapillary level [41,42]. This is mediated by the axonal reflex, an endothelium-derived hyperpolarizing factor.

Tibiriça et al. [58] observed that capillary recruitment during PORH (% increase in mean capillary density) in fingers was significantly higher in controls than in diabetic patients without disease complications. Meanwhile, Forst et al. [70] showed that type 1 diabetics with peripheral neuropathy have no significant changes in capillary blood flow. In our study, we observed that capillary density after the PORH test was significantly lower in diabetic young patients, with no significant differences between the D1 and D2 groups. In contrast, both in the diabetic group as a whole and in patients with shorter disease duration, we observed a significant decrease in capillary density during the PORH test. This suggests that young patients with T1D who do not have microangiopathic complications have a smaller capillary reserve.

We have also previously described skin microcirculatory reactivity in response to the application of the PORH test in a group of young patients with T1D [85].

Our previous study showed that cutaneous microvascular reactivity, assessed by capillaroscopy and PORH, was significantly lower in patients with diabetes and autoimmune thyroiditis than in patients with diabetes alone [85]. Therefore, we decided to exclude patients with Hashimoto disease from the study. The effect of thyroid dysfunction on skin microcirculation has also been demonstrated by Pazos-Moura et al. [86], Weiss et al. [14], and Bajuk et al. [87]. In addition, hormonal changes associated with puberty may affect skin microcirculation [88].

### 4.4. Limitations

However, a natural limitation of this study is its cross-sectional nature. A prospective study analyzing data collected over several years of disease course would further allow us to establish the value of the non-invasive methods we used in detecting early signs of microcirculatory dysfunction. Another limitation of the project is undoubtedly the lack of accurate determination of sex hormone levels during puberty, the levels of which may affect endothelial function. However, we did include sexual maturity according to Tanner’s scale.

## 5. Conclusions

In a group of young diabetic patients, we demonstrated that capillaroscopy with the use of PORH, but not TcPO_2_, may allow to detection of microcirculatory dysfunction in patients with type 1 diabetes of variable duration. This was independent of patient age, lipid levels, and thyroid hormone levels.

In our study group of young patients with uncomplicated type 1 diabetes for up to 15 years, neither capillaroscopy nor TcPO_2_ showed any differences in the microvascular parameters within the group.

The results of our study confirm that in patients with a shorter duration of disease, simultaneous examination of multiple sites provides allows for a more complete characterization of microcirculatory structure and function, as the dysfunction may not be simultaneously present in all tests. However, in patients with a more prolonged disease course, peripheral microcirculation disturbances may be identified by examining even a single vascular area, such as nail bed capillaries or transcutaneous oxygen pressure.

## Figures and Tables

**Table 1:** Characteristics of all diabetic patients enrolled in the study as well as both of the subgroups. divided according to the median of T1D duration, and healthy controls. Data are presented as median (range) or mean ± SD.

Characteristics	Control Group, C N = 28	Diabetic Patients, D N = 67	*p* for between Group Comparison	Diabetic Patients Subgroups According to the Median T1D Duration		*p*	Post Hoc Comparison		
				<4.65 y.	≥4.65 y.		C vs. D1	C vs. D2	D1 vs. D2
				D1: (N = 33)	D2: (N = 34)				
Males n (%)	54% (15)	29 (43)	0.36	48/52	38/62	0.46			
BMI [kg/m^2^]	19.3 (14.4–24.7)	20.2 (14.5–29.7)	0.43	20 (15–29.7)	20.8 (14.5–27.4)	0.13			
Age, [years]	14.8 (11.3–17.7) 14.5 ± 2	14.6 (10.9–18) 14.9 ±1.9	0.49	14 (10.9–17.8) 14.1 ± 1.7	16.1 (11.7–18) 15.7 ± 1.9	0.003	1	0.07	0.003
Onset of diabetes [age]		10.3 (2.1–15.0)	na	11.6 (6.9–15)	8.5 (2.0–12.7)	<0.001			<0.001
T1D duration [years]		4.65 (0.6–14.5)	na	2.38 (0.55–4.65)	7.3 (4.62–14.5)	<0.001			<0.001
Insulin dose units/24 h		41.5 (10.3–100)	na	36 (10–57)	47.5 (30–100)	0.63			
Time of pump treatment in ratio to T1D duration [%]		35 (0–97)	na	0 (0–93)	36 (0–97)	0.13			
HbA_1c_ [%]	5.3 (4.8–5.6)	7.6 (5.3–13.6)		7.1 (5.3–11)	7.6 (6.2–13.6)	<0.001	<0.001	<0.001	0.86
Episodes of mild hypoglycemia [N/last Month]		6 (0–30)	na	4 (0–30)	10 (0–20)	0.02			0.02
Episodes of severe hypoglycemia [N/last year]		0 (0–3)	na	0 (0–3)	0 (0–2)	0.77			
Systolic blood pressure [mmHg]	109 ± 9	106 ± 11	0.21	104 ± 12	108 ± 10	0.1			
Diastolic blood pressure [mmHg]	62 ± 5	60 ± 6	0.12	59 ± 6	60 ± 6	0.25			
Heart rate [beats/min.]	79 ± 12	80 ± 10	0.59	79 ± 10	80 ± 10	0.82			

The value of *p* < 0.05 was regarded as statistically significant. Abbreviations: HbA1c—glycated hemoglobin; BMI—body mass index; T1D—diabetes mellitus; na—not applicable.

**Table 2:** Comparison of laboratory results in all diabetic patients, diabetic patients divided into subgroups (D1, D2) and healthy controls (C). Data are presented as median (range).

Characteristics	Control Group, C N = 28	Diabetic Patients, D N = 67	*p* for between Groups Comparison	Diabetic Patients Subgroups According to the Median T1D Duration		*p*	Post Hoc Comparison		
				<4.65 y	≥4.65 y		C vs. D1	C vs. D2	D1 vs. D2
				D1: (N = 33)	D2: (N = 34)				
Total cholesterol [mg/dL]	163 (120–218)	178 (119–288)	0.004	169 (119–228)	183 (125–288)	0.007	0.23	0.005	0.72
Cholesterol LDL [mg/dL]	95 (64–127)	102 (61–188)	0.11	90.5 (61–131)	108 (70–188)	0.008	1	0.03	0.03
Cholesterol HDL [mg/dL]	51 (41–82)	55 (33–120)	0.21	58.5 (33–120)	51 (35–95)	0.07			
Triglycerides [mg/dL]	54 (36–117)	70 (37–294)	0.003	61.5 (37–153)	76 (46–294)	<0.001	0.8	<0.001	0.04
Serum creatinine [mg/dL]	0.64 (0.52–0.94)	0.69 (0.46–0.95)	0.3	0.7 (0.5–0.9)	0.7 (0.5–1)	0.34			
Albuminuria [mg/24 h]	Not tested	6.4 (2.5–27)	n.a.	7 (2.5–24.1)	5 (2.5–27)	n.a	n.a.	n.a.	0.36
TSH [mIU/L]	1.2 (0.58–2.37)	1.7 (0.6–4)	0.001	1.6 (0.7–3.6)	1.8 (0.6–4)	0.006	0.04	0.008	1
fT4 [pmol/L]	13.6 (11.4–16.8)	12.4 (9–15)	0.001	12.5 (10–14.7)	12.2 (9–15)	0.004	0.03	0.006	1
C-reactive protein [mg/L]	0.2 (0.1–2.9)	0.4 (0.1–8.5)	0.14	0.6 (0.1–3.7)	0.4 (0.1–8.5)	0.33			

The value of *p* < 0.05 was regarded as statistically significant. Abbreviations: TSH: thyroid-stimulating hormone; fT4: free thyroxine; LDL—low-density lipoproteins; HDL—high-density lipoproteins, n.a.—not applicable.

**Table 3:** Comparison of microcirculation parameters in all diabetic patients, diabetic patients divided into subgroups (D1, D2) and healthy controls (C). Data are presented as median (range).

Characteristics	Control Group, C N = 28	Diabetic Patients, D N = 67	*p* for between-Group Comparison	Diabetic Patients Subgroups According to the Median T1D Duration		*p*	Post Hoc Comparison		
				<4.65 y.	≥4.65 y.		C vs. D1	C vs. D2	D1 vs. D2
				D1: (N = 33)	D2: (N = 34)				
Capillaroscopy									
coverage_BASE_ [%]	18.7 (14.2–6.4)	17.5 (11.5–24.8)	0.02	17.5 (11.5–22.3)	17.3(12.9–24.8)	0.08			
After adjustment for: age age. lipid profile and thyroid hormones						0.07			
						0.009	0.04	0.06	0.72
coverage_PORH_ [%]	17.9 (13.7–23.2)	16.2 (9.8–24.4) # (*p* < 0.001)	<0.001	15.5 (9.8–22.3) # (*p* = 0.001)	16.7(10.3–24.4)	0.001	<0.001	0.06	0.53
After adjustment for: age age. lipid profile and thyroid hormones						0.004	<0.001	0.04	0.16
						<0.001	0.002	0.03	0.20
∆Coverage_PB_ [%]	−0.4 (−6.4–5.4)	−1.1 (−7.3–4.9)	0.47	−0.8 (−7.2–2.3)	−1.2(−7.3–4.9)	0.54			
After adjustment for: age age. lipid profile and thyroid hormones						0.28			
						0.50			
Capillary reactivity	−2.4 (−24.2–35)	−5.5 (−35–31.3)	0.40	−5.3 (−35.1–14.8)	−6.5(−34–31.3)	0.51			
After adjustment for: age age. lipid profile and thyroid hormones						0.18			
						0.37			
Distance_BASE_ [µm]	218.3 (177.5–278)	224.3 (165.8–377.7)	0.35	220.8 (165.8–377.7)	230.4(179.4–263.9)	0.29			
After adjustment for: age age. lipid profile and thyroid hormones						0.73			
						0.23			
Distance_PORH_ [µm]	228.2 (167.6–294)	232.8 (166.3–345) # (*p* = 0.04)	0.19	232.9 (166.3–319.8)	232.7(180.5–345.1)	0.43			
After adjustment for: age age. lipid profile and thyroid hormones						0.29			
						0.09			
∆DistancePB [µm]	6.4 (−52.3–69.2)	9.6 (−70.4–90.5)	0.59	10.8 (−59–74)	6.9(−70.4–90.5)	0.75			
After adjustment for: age age. lipid profile and thyroid hormones						0.69			
						0.84			
The transcutaneous oxygen pressure									
TcPO_2__baza [mmHg]	56.3 (18.6–81.1)	51.7 (27.9–80.8)	0.16	54.1 (27.9–71.5)	51.3(30–81)	0.28			
After adjustment for: age age. lipid profile and thyroid hormones						0.32			
						0.50			
TcPO_2__zero [mmHg]	1.6 (0.4–12.0)	2.3 (0.7–18.8)	0.007	2.01 (0.7–18.8)	2.7(1.0–9)	<0.001	0.86	<0.001	0.02
After adjustment for: age age. lipid profile and thyroid hormones						0.30			
						0.30			
TTR [s]	85 (41–240)	83 (42–240)	0.96	85 (42–240)	80 (42–240)	0.99			
After adjustment for: age age. lipid profile and thyroid hormones						0.99			
						0.83			
SLOPE	0.61 (0.15–1.32)	0.57 (0.14–1.13)	0.36	0.56 (0.1–1.13)	0.59(0.2–1.1)	0.53			
After adjustment for: age age. lipid profile and thyroid hormones						0.22			
						0.71			

The value of *p* < 0.05 was regarded as statistically significant. # indicates the value of statistical significance obtained for comparing pre-post values of PORH. Abbreviations: T1D—diabetes mellitus; PORH-post-reactive hyperemia; Coverage_BASE_—the ratio of the capillary area to the total area of the determined rows in the baseline condition; Coverage_PORH_—the ratio of capillary area to the total area of the determined rows after PORH; ∆Coverage_PB_ = Coverage_PORH_ − Coverage_BASE_—the difference between the values of coverage obtained after and before PORH; Capillary reactivity = ∆CoveragePB/CoverageBASE; DistanceBASE—mean distance between successive capillaries in baseline condition; Distance_PORH_—average distance between successive capillaries after PORH; ∆Distance_PB_ = Distance_PORH_ − Distance_BASE_—the difference between the distance values obtained after and before PORH.

## Data Availability

The research data can be requested from the first author.
